# Improved random coincidence estimation including triple coincidence detection in PET

**DOI:** 10.3389/fnume.2026.1739575

**Published:** 2026-03-05

**Authors:** Debora Niekämper, Jürgen J. Scheins, Elisabeth Pfaehler, Joaquin L. Herraiz, N. Jon Shah, Christoph Lerche

**Affiliations:** 1 Institute for Neuroscience and Medicine (INM-4), Forschungszentrum Jülich GmbH, Jülich, Germany; 2 Department of Physics, RWTH Aachen University, Aachen, Germany; 3 Nuclear Physics Group, EMFTEL and IPARCOS, Complutense University of Madrid, Madrid, Spain; 4 Institute of Neuroscience and Medicine 11 (INM-11), Forschungszentrum Jülich GmbH, Jülich, Germany; 5 JARA-Brain-Translational Medicine, Aachen, Germany; 6 Department of Neurology, RWTH Aachen University, Aachen, Germany

**Keywords:** coincidence identification, delayed window method, positron emission tomography, random estimation, triple coincidences

## Abstract

Coincidence detection in PET is inherently prone to misidentification due to the presence of randomly occurring singles from different decays within the coincidence time window. Random triple coincidences, arising when three singles from at least two decays are detected within this window, can lead to bias by acceptance without further consideration in the double coincidence identification, or result in sensitivity loss if rejected. True triple coincidences, which occur with $\beta^{+}\text{-}\gamma$-emitters used in dual-tracer PET and positronium lifetime imaging, are also affected by random coincidences, leading to errors that necessitate appropriate correction. The aim of this work was to develop an accurate method for estimating random double and triple coincidences which is crucial for quantitative PET imaging of $\beta^{+}-$ and $\beta^{+}\text{-}\gamma$-emitters. The number of random triple coincidences was evaluated for both emitter types. To process double and triple coincidences separately, coincidence identification schemes were defined with intervals free of other singles as vetoes for accepted coincidences. Random coincidences were estimated using extended delayed window techniques, which match the interval sizes for coincidence windows and vetoes. Coincidences comprising singles from two and three decays were separated in simulation studies, and two delayed windows were applied to guarantee the singles’ independence in the latter case. Correction factors from additional coincidence identification schemes were used to compensate for differences in the total veto interval size between the prompt and delayed methods. The methods were evaluated using simulations for different isotopes, coincidence windows, phantom shapes, and activities. The simulation results were then validated against measurements obtained with a brain PET scanner. The total random coincidence rate for the entire scanner was estimated with a relative deviation of <3% for double coincidences and <5% for triple coincidences of two decays of $\beta^{+}$-emitters and $\beta^{+}\text{-}\gamma$-emitters for the investigated coincidence identification schemes and for the simulated cases.

## 1 Introduction

Positron emission tomography (PET) is an imaging modality based on the coincident detection of photon pairs created when positrons emitted by radiotracers annihilate in the surrounding tissue [1]. While conventionally limited to one tracer per measurement, recently proposed dual-tracer PET methods combine a pure $\beta^{+}$-emitter, such as ${}^{11}C$, and a $\beta^{+}\text{-}\gamma$-emitter, such as ${}^{68}\mathrm{Ge}$ or ${}^{22}\mathrm{Na}$, for which an additional photon ($\gamma_{\text{\textbackslash{},prompt}}$) arises in the decay process. This enables the simultaneous study of different metabolically active molecules involved in the same biological processes [2–4].

**Figure 1:**
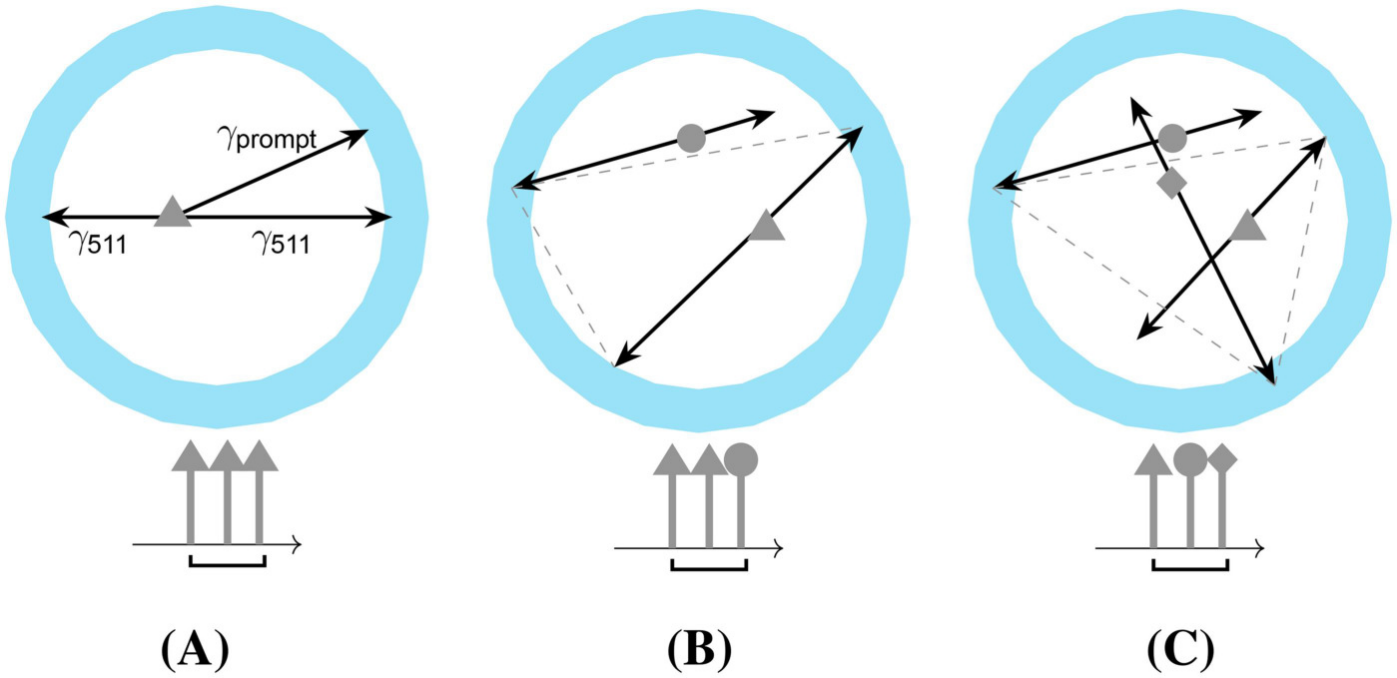
Different types of triples: **(A)** true triple, **(B)** random coincidence by photons from two different decays (*two-decay triple*), and **(C)** random coincidence by photons from three different decays (*three-decay triple*).

In order to separate the two tracers utilizing the $\gamma_{\text{\textbackslash{},prompt}}$, the coincidence processing of *doubles*, i.e., combinations of two *singles*, is extended to *triples* (Figure 1), i.e., combinations of three *singles*. If the energy of the $\gamma_{\text{\textbackslash{},prompt}}$ is higher than that of the annihilation photons, it can be used as a tag to mark the coincidences belonging to the $\beta^{+}\text{-}\gamma$-tracer. An additional application for triples processing is positronium lifetime imaging. This technique is based on positron decay via the formation of positronium [5]. As the detection of a $\gamma_{\text{\textbackslash{},prompt}}$ indicates the time at which the decay occurred, the ortho-positronium lifetime can be determined by measuring the time delay between the detection of the $\gamma_{\text{\textbackslash{},prompt}}$ and the annihilation photon pair using the time of flight information. The lifetime contains information about the microenvironmental material [6–9].

**Figure 2:**
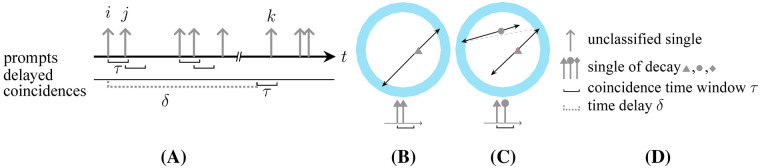
General naming and representation convention of coincidences in a list of single events. The association with the different decays is represented by the different line caps and colors. **(A)** Prompt and delayed coincidence identification, identified **(B)** true, and **(C)** random, and **(D)** legend.

In most PET systems, coincidences are typically identified by hardware during acquisition. However, in more recent systems, coincidences can be identified by software. This is achieved by creating a chronologically sorted list of qualified singles that meet specific energy criteria and then searching for single combinations within a coincidence time window, τ (Figure 2A). The coincidences found within τ are referred to as *prompts*, which consist of *trues* comprising singles from the same decay (Figure 2B), and *randoms*, which are combinations of singles from different decays (Figure 2C). The legend for the sketches is provided in Figure 2D.

Randoms lead to spuriously increased background in the reconstructed images, requiring appropriate correction methods for quantitative results. One of the two commonly utilized approaches for the estimation of random double coincidence rates (R) is the singles rate method [10, 11]. This method is based on the singles rates $S_{g}$ and $S_{h}$ measured in detectors g and h and combined by Equation 1:

$$R_{\mathrm{gh}}\approx 2\tau S_{g}S_{h}.$$ (1)

The second approach is the delayed window technique [10]. In this approach, *delayed coincidences*, i.e., combinations with coincident singles within a delayed time window, are counted. This time window is shifted by an arbitrary delay (δ) (Figure 2A, last row), with a lower limit sufficient so as not to detect trues, and an upper limit defined by the necessity that the activity remains unchanged. Studies comparing these two approaches demonstrate a lower accuracy for the first and a higher noise level for the second approach [12, 28]. To address these disadvantages, methods with improved singles rate modeling [14, 15] and variance reduction methods have been proposed [16, 17].

When a single i occurs at time $t_{i}$ and more than one single j, or in the case of triples, more than two singles fulfil the coincidence condition $|t_{j}-t_{i}|<\tau$, various strategies can be employed to treat these *multiples* [15, 18]. However, while the rejection of multiples leads to sensitivity loss, acceptance can cause bias in quantification [19]. The impact of different coincidence policies [13, 20] and the number of multiples has been previously explored [21–23] and options to reduce their number with an additional geometrical selection or a second variable coincidence time window have been suggested [24].

Triples and their corrections have been explored in the context of inter-crystal scatter, where lowering the energy window can lead to a sensitivity gain [25, 26]. They have also been investigated with regard to random estimation [27–29]. Other studies on triples focus on selecting the most probable double combination within a triple [30] and the identification of the annihilation pair for $\beta^{+}\text{-}\gamma$-emitters [31].

For pure $\beta^{+}$-emitters and $\beta^{+}\text{-}\gamma$-emitters, random triples are composed of singles originating from either two (*two-decay triple*, Figure 1B) or, less commonly, three (*three-decay triple*, Figure 1C) different decays. The impact of dead time on random rates has been investigated by Robinson et al. [32]. Recently, Moore et al. [33] demonstrated improved image quality for dual-tracer PET by implementing the first random correction approaches, which estimate random triples using a scaled double image, with the scaling factor determined by a fraction from singles and doubles rates. Huang et al. [34] introduce a random estimation method for positronium lifetime image reconstruction based on multiple delayed coincidence windows. However, the accuracy and precision of random triple estimation by multiple delayed coincidence windows remain unclear.

The objective of this work is to establish methods for the accurate random estimation of doubles and triples for $\beta^{+}$-emitters and $\beta^{+}\text{-}\gamma$-emitters and to evaluate their accuracy and precision by Monte Carlo (MC) simulations. As part of this work, the delayed window technique was adapted to enable different definitions of double coincidence to be considered and enable triple random rates to be estimated.

## 2 Methods

The goal of this study is to develop and validate methods for random coincidence correction involving triples. To achieve this, different approaches for coincidence identification within the prompt window were first designed. Methods were then established to estimate the random double contamination, followed by techniques for assessing random triple contamination arising from one or two decays in $\beta^{+}$- and $\beta^{+}\text{-}\gamma$-emitters. Random and delayed coincidence rates were modeled to develop methods for estimating random coincidences. However, modeling is not necessary when applying these methods to simulated or measured data. When applying the methods, it is only necessary to identify delayed coincidences with specific settings and potentially combining the determined rates. A summary of the applied naming conventions can be found in Glossary.

### 2.1 Coincidence identification and ambiguities

Two common approaches to opening coincidence time windows are the multiple-window method, in which each single opens a time window, and the single-window method, in which a single only opens a coincidence time window if it is not part of a prior time window. In both approaches, if more than two singles are identified to be in coincidence, the possible treatments include the following: (1) discarding the coincidence event; (2) selecting one of the possible pairs based on additional information, such as energy and location; (3) assigning the singles to coincidences with their direct predecessor and successor; or (4) creating coincidences from all possible combinations of coincident singles [18].

In this study, the multiple-window approach was used for a preselection of coincidences (lower time windows underneath the time arrows in the sketches below). In addition, *veto schemes* were applied, i.e., time intervals that were required to be free of singles were created around the timestamps of the considered singles (hatched area and upper time windows above the single tips in the sketches for $V_{\text{inner}}$ and $V_{\text{outer}}$ below). The coincidences were rejected if another single is found in the intervals. The following *veto schemes* were applied to the list of identified coincidences, with i denoting the initial single of the considered coincidence:

**Table:** 

$V_{\text{no}}$	No veto windows. All singles are combined into coincidences, leading to three double combinations of three singles in a window and allowing combinations with other singles around the window	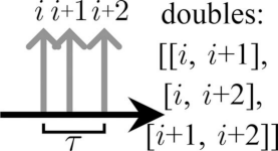 .
$V_{\text{between}}$	Veto windows between singles. Singles are combined into coincidences with their neighboring singles. Combinations of coincident singles are excluded if another single is found in between. This approach leads to two coincidences for three singles in a window	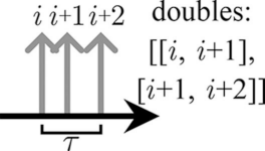 .
$V_{\text{inner}}$	Inner veto windows. Coincidences are excluded if another single is found in a window with both coincident singles. Thus, the conditions for acceptance are $t_{i+2}-t_{i}>\tau$ and $t_{i+1}-t_{i-1}>\tau$	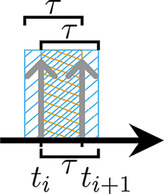 .
$V_{\text{outer}}$	Outer veto windows. Coincidences are excluded if at least one of the singles is in coincidence with an additional single. Thus, the conditions for acceptance are $t_{i+2}-t_{i+1}>\tau$ and $t_{i}-t_{i-1}>\tau$	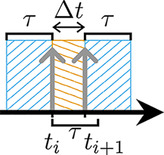 .

Throughout this work, coincidences formed by m singles within the coincidence time window are referred to as multiples of order m. The latter veto schemes are extended for multiples by initiating the veto windows at the timestamps of the first and last single in the group of m singles.

### 2.2 Classification and rate estimation of random double coincidences

Each detected single can be assigned to one of two groups depending on whether the opposite photon from its annihilation pair was detected or not. The former case is termed a *paired single* throughout this work, and the latter case an *unpaired single*. Therefore, the following classes of random doubles are obtained: comprising two *unpaired singles*, two *paired singles*, and one of each type, respectively. Veto scheme $V_{\text{outer}}$ only accepts randoms of two *unpaired singles*. Therefore, for an ideal point source, the random double rate $R_{\text{outer}}^{\text{( d) }}$ for $V_{\text{outer}}$ over the whole scanner is approximately given Equation 2a:

$$R_{\text{outer}}^{\text{( d) }}\approx \underset{\text{single that opened window}}{\underbrace{\epsilon_{1}A}}\overset{\text{no single before}}{\overbrace{\exp(-\tau \epsilon_{\geq 1}A)}}\underset{\text{single in window}}{\underbrace{\epsilon_{1}A\tau }}\overset{\text{no single between}}{\overbrace{\exp(-\epsilon_{\geq 1}A\langle \Delta t\rangle )}}\underset{\text{no single after}}{\underbrace{\exp(-\epsilon_{\geq 1}A\tau )}}$$ (2a)

$$=\tau {\left(\epsilon_{1}A\right)}^{2}\exp(-\epsilon_{\geq 1}A\left(2\tau +\langle \Delta t\rangle \right))$$ (2b)

with A representing the activity, $\epsilon_{\geq 1}$ representing the efficiency for detecting at least one photon, $\epsilon_{1}$ the efficiency for detecting exactly one photon, and ⟨Δt⟩ (≈0.5τ, where this value was obtained assuming a uniform distribution and which was verified in the simulations) representing the expected time difference between singles of accepted coincidences. This equation can be derived similarly to the formulas introduced in Oliver and Rafecas [14] for improving the random rates method based on Poisson distributions of the singles rates. An adjustment is made for the veto interval size and the reduction of the singles rates to *unpaired singles*, since the veto scheme $V_{\text{outer}}$ rejects randoms containing *paired singles*. It should be noted that the efficiencies $\epsilon_{1}$ and $\epsilon_{\geq 1}$ are energy dependent. Therefore, they are, in general, slightly different for scattered and unscattered photons or for prompt γ-photons (in some isotopes). However, this effect is not considered in this work for simplicity.

The random estimation has to be in accordance with the selected coincidence identification scheme. Therefore, window schemes for identifying delayed coincidences are introduced that, when adequately combined, yield the same counting probability for accidental coincidences as the schemes applied to the prompt window in the simulated data.

**Figure 3:**
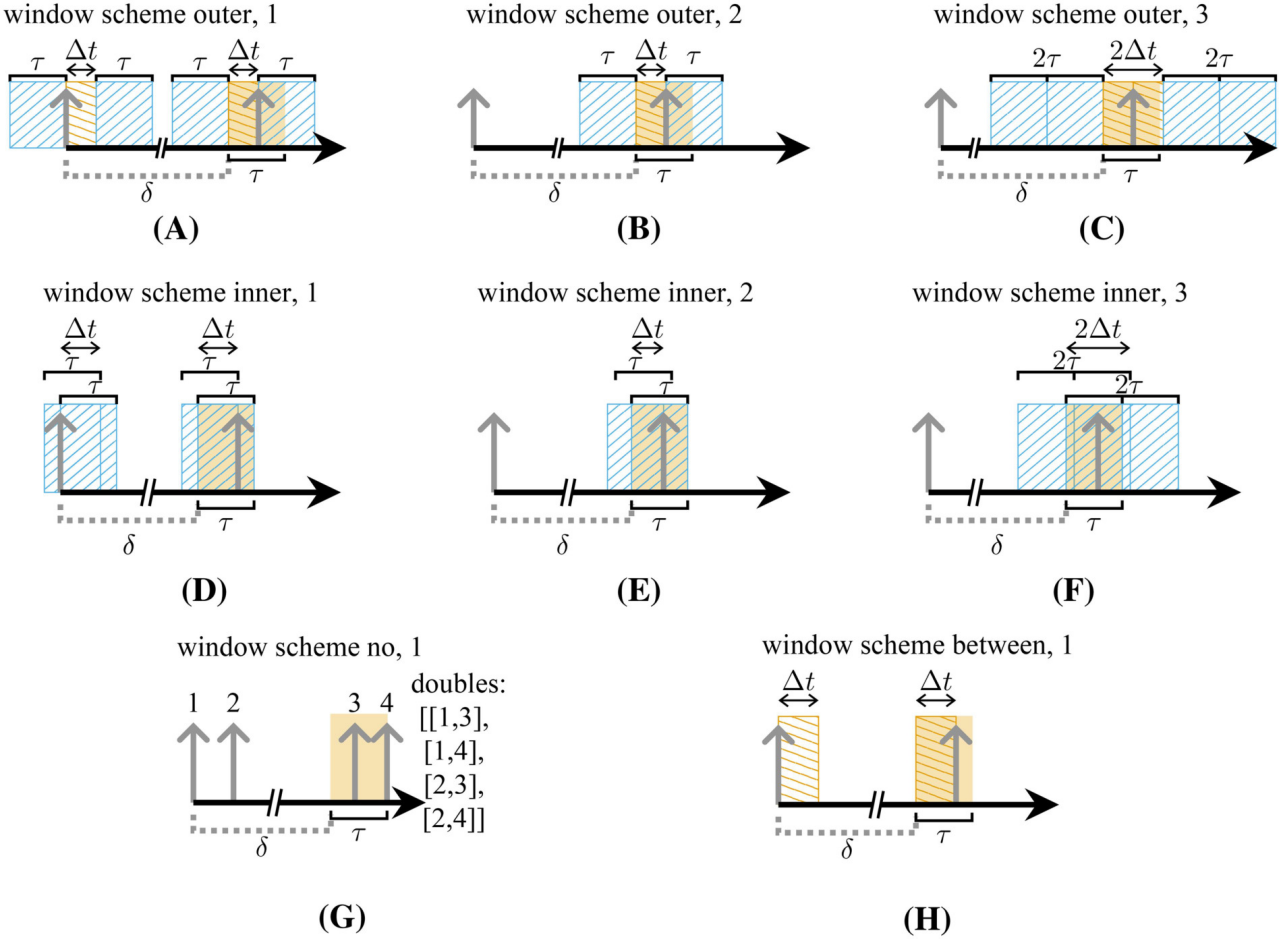
Evaluated window schemes for counting delayed doubles. First row **(A–C)**: for veto scheme $V_{\text{outer}}$; second row **(D–F)**: $V_{\text{inner}}$, **(G)** $V_{\text{no}}$, and **(H)** $V_{\text{between}}$. The search area is highlighted in orange and the veto intervals are hatched.

For the delayed coincidences selection, veto windows were applied around both singles to only allow contributions from *unpaired singles* (Figure 3A, window scheme 1). For an appropriate estimation, the veto window size of prompts and delayed coincidences was set to be equal and a necessary scaling factor was applied. The scaling factor can, for example, be provided by the ratio of rates determined by window schemes 2 and 3 (Figures 3B, C). Both window schemes apply veto windows around either the first or the second single, but for window scheme 3, the veto interval is doubled.

The delayed double rates $D_{\text{outer},l}^{\text{( d) }}$ for the window schemes l∈{1,2,3} can be approximately described by

$$D_{\text{outer, 1}}^{\text{( d) }}=\tau {\left(\epsilon_{1}A\right)}^{2}\exp\left(-2\epsilon_{\geq 1}\left(2\tau +\langle \Delta t\rangle \right)A\right)$$ (3a)

$$D_{\text{outer, 2}}^{\text{( d) }}=\tau \epsilon_{1}\left(\epsilon_{1}+2\epsilon_{2}\right)A^{2}\exp\left(-\epsilon_{\geq 1}\left(2\tau +\langle \Delta t\rangle \right)A\right)$$ (3b)

$$D_{\text{outer, 3}}^{\text{( d) }}=\tau \epsilon_{1}\left(\epsilon_{1}+2\epsilon_{2}\right)A^{2}\exp\left(-2\epsilon_{\geq 1}\left(2\tau +\langle \Delta t\rangle \right)A\right),$$ (3c)

with $\epsilon_{2}$ denoting the detection efficiency of the annihilation photon pair. Following from Equations 3a–3c, the pure random double rate from Equation 2b can be estimated by combining the rates determined with the three window schemes (Figures 3A–C):

$${\hat{R}}_{\text{outer}}^{\text{( d) }}=\frac{D_{\text{outer, 1}}^{\text{( d) }}D_{\text{outer, 2}}^{\text{( d) }}}{D_{\text{outer, 3}}^{\text{( d) }}},$$ (3d)

where ${\hat{R}}_{\text{outer}}^{\text{( d) }}$ denotes the estimate for the corresponding quantity $R_{\text{outer}}^{\text{( d) }}$. The applied estimation for $V_{\text{inner}}$ is consistent with the estimation given in Equation 3d by replacing the exponential argument with (2τ−⟨Δt⟩). Here, a small fraction of randoms containing paired singles are ignored. These are also included in $D_{\text{inner, 1}}^{\text{( d) }}$, but there they would require a lower correction factor. This is because only coincidences of singles of a true with other singles in an interval of size $\Delta t_{\text{true}}$ at the end of the coincidence time window are not rejected, leading to an expectation value higher than ⟨Δt⟩.

We approximate the random rates for $V_{\text{no}}$ and $V_{\text{between}}$ by

$$R_{\text{no}}^{\text{( d) }}\approx {\left(\epsilon_{1}+2\epsilon_{2}\right)}^{2}\tau A^{2}$$ (4)

$$R_{\text{between}}^{\text{( d) }}\approx {\left(\epsilon_{1}+\epsilon_{2}\right)}^{2}\tau A^{2}\exp\left(-\epsilon_{\geq 1}\langle \Delta t\rangle A\right).$$ (5)

For $V_{\text{no}}$, the initial single is combined with all singles in the delayed time window without applying any veto windows. For $V_{\text{between}}$, the following two veto intervals are applied: one starting from the onset of the delayed window until $t_{k}$ and another one of the same length starting at time $t_{i}$. We assume that $D_{\text{no}}^{\text{( d) }}\approx R_{\text{no}}^{\text{( d) }}$ and $D_{\text{between}}^{\text{( d) }}\approx R_{\text{between}}^{\text{( d) }}\exp(-\epsilon_{\geq 1}\langle \Delta t\rangle A)$. In the latter case, the difference between the randoms and the delayed coincidences is caused by the doubled veto interval. Omitting the interval would result in a higher deviation by allowing coincidence rates $(\epsilon_{1}+2\epsilon_{2})A$ in the window.

### 2.3 Rate estimation for random triples

#### 2.3.1 Delayed coincidence time windows for two- and three-decay random triples

**Figure 4:**
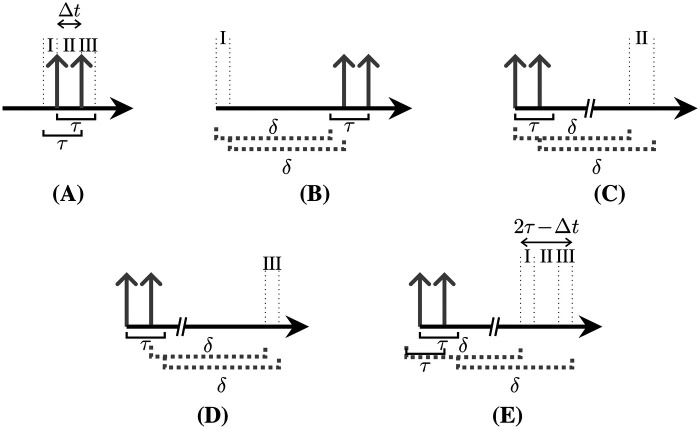
All possible time window intervals for the coincidence detection of the third single of a random triple. The additional single can be located before (I), between (II), or after (III) a double **(A)**. The intervals can be covered by separated search window schemes **(B)**–**(D)** or simultaneously **(E)**.

There are three different scenarios for a two-decay random triple in a prompt window, namely, the additional single can be detected before (I), between (II), or after (III) the true double (Figure 4A). These scenarios can be replicated for delayed coincidences by searching I: a double in a window of lengths τ after the occurrence of the single (Figure 4B); II: a double and an additional single in a delayed window of size Δt (Figure 4C); III: a double and an additional single in a delayed window of size τ−Δt (Figure 4D). These scenarios can be summarized by searching for a double {i,j} and an additional single k in a window of size $2\tau -\Delta t_{i,j}$.

For estimating the contained three-decay triples, a similar window scheme with the same window length is used. However, a further delay is introduced between the first two singles, and $\Delta t_{i,j}$ is set to $t_{j}-\delta$.

We estimate the three decay random triples with two delayed windows of size τ. The determined rate must be halved, since the squared probability of finding one event in a window is twice the probability of finding two events in a window.

**Figure 5:**
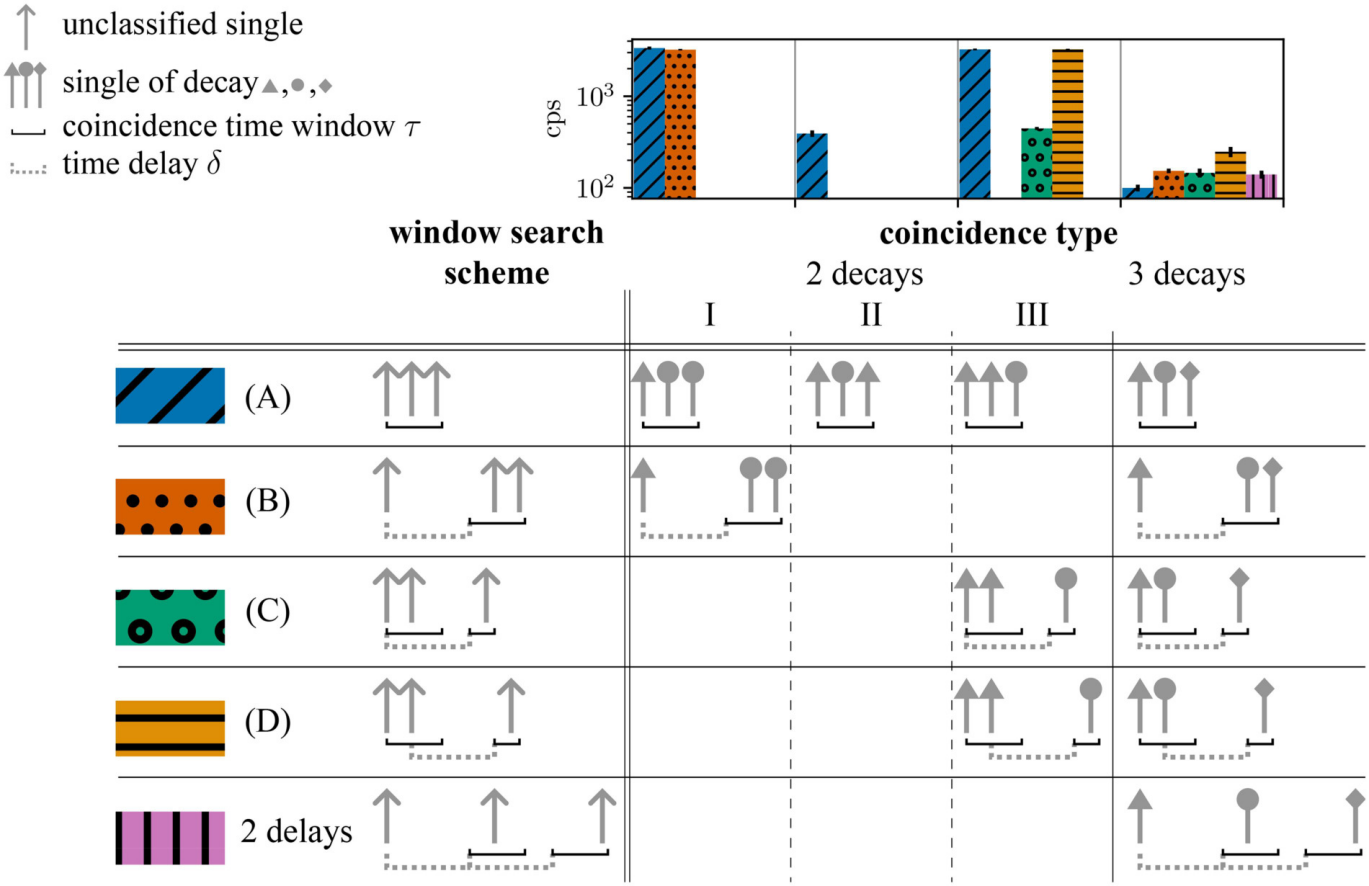
Histogram (upper part of figure) of detected random triples with the standard error and a table illustrating the different window schemes and coincidence type classifications. Columns 3 to 6 represent the different types of triple coincidence, namely, three-decay and two-decay triples, where the two-decay triples are further differentiated into groups I–III according to the position of the single from the additional decay in the triple (as indicated by the different arrowheads). The rows are used to differentiate between the window search schemes, which are visualized schematically in the second column. The cases in the first four rows correspond to the window intervals shown in detail in Figures 4A–D.

To illustrate which contributions can be identified with which window scheme, Figure 5 shows the different contributions to random triples for a $\beta^{+}$-emitter determined with different window schemes. The count rates are shown alongside a column-aligned table that displays the different contributions. In this table, each row represents a specific window scheme. Furthermore, the corresponding color encoding used in the count rate histograms is indicated. The first three columns of the table represent two-decay random triples distinguished by the position of the additional single and corresponding to cases I–III in the prompt window. The final column represents three-decay triples.

#### 2.3.2 Rate estimation for two-decay random triples of $\beta^{+}$-emitters identified with $V_{\text{outer}}$

**Figure 6:**
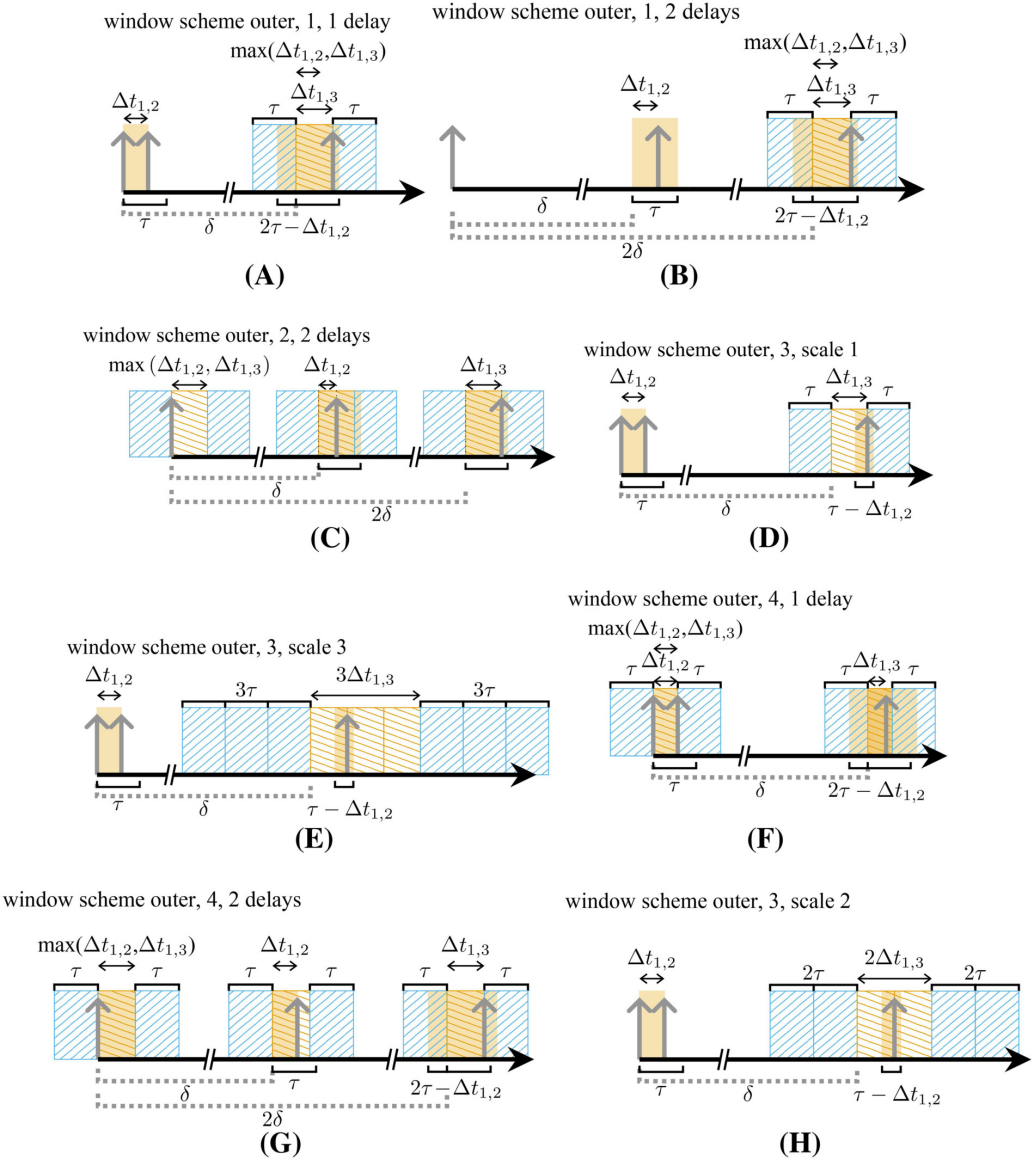
Window schemes for the estimation of random triple rates of $V_{\text{outer}}$. **(A)** and **(B)** for two-decay triples of $\beta^{+}$-emitters; **(C)**–**(E)** for three-decay triples; and **(F)**, **(H)**, and **(C)** for two-decay triples for $\beta^{+}\text{-}\gamma$-emitters.

For $V_{\text{outer}}$, the rate of two-decay triples is given by

$$R_{\text{outer}}^{\text{( t) , 2decays}}\approx \epsilon_{2}\epsilon_{1}\left(2\tau -\langle \Delta t_{\text{true}}\rangle \right)A^{2}\exp\left(-\epsilon_{\geq 1}\left(2\tau +\langle \Delta t_{\text{1, 3}}^{\text{2decays}}\rangle \right)A\right),$$ (6)

with the expected time difference between both singles of a true $\left\langle \Delta t_{\text{true}}\right\rangle$ and the expected time difference between the first and the last single of the triple $\left\langle \Delta t_{\text{1, 3}}^{\text{2decays}}\right\rangle$. In this case, the veto window size for the delayed coincidences matching the prompt window is $2\tau +\max(|t_{k}-\delta |,\Delta t_{i,i+1})$. The veto window can be either applied around the included double, allowing additional contributions with efficiencies $\epsilon_{1}\epsilon_{2}$ or contributions with $\epsilon_{\geq 1}\epsilon_{1}^{2}+\epsilon_{2}^{2}$, or it can be applied around the delayed single, allowing additional contributions with efficiencies $\epsilon_{1}\epsilon_{\geq 1}\epsilon_{\geq 1}$. Since the first option additionally accepts randoms of paired singles, the latter option was chosen (Figure 6A, rate $D_{\text{outer, 1}}^{\text{( t) , 1delay}}$ with subscript 1 indicating scheme 1 and superscript 1delay indicating one delayed window). The number of additional contributions can be estimated with a scheme with the same veto windows, but it requires a further delay between the first two singles to guarantee combinations of three uncorrelated singles (Figure 6B, $\text{rate}\;D_{\text{outer, 1}}^{\text{( t) , 2delays}}$). Thus, the randoms are estimated by identifying triples, employing the scheme in Figure 4E, using one and two delays and by subtracting the latter ones:

$${\hat{R}}_{\text{outer},a}^{\text{( t) , 2decays}}=D_{\text{outer, 1}}^{\text{( t) , 1delay}}-D_{\text{outer, 1}}^{\text{( t) , 2delays}},$$ (7)

with a in the subscript indicating method a.

#### 2.3.3 Rate estimation for three-decay random triples identified with $V_{\text{outer}}$

For the case $V_{\text{outer}}$, two delays can also be used to estimate the number of three-decay random triples and, to exclusively select triples of unpaired singles, veto windows are applied around all three singles. As with the previous approach for doubles, a scaling factor for the tripled veto window size needs to be applied. This factor can be determined by the ratio of triples identified with window schemes 3 and scale 1 and 3 (Figures 6D, E). According to the Poisson probability to find two events in the opened windows, the triples random rate of three independent decays can be approximated by

$$R_{\text{outer}}^{\text{( t) , 3decays}}=\frac{1}{2}\epsilon_{1}^{3}\tau^{2}A^{3}\exp\left(-\epsilon_{\geq 1}\left(2\tau +\langle \Delta t_{1,3}^{\text{3decays}}\rangle \right)A\right)$$ (8)

and estimated in analogy to Equation 3d by

$${\hat{R}}_{\mathrm{outer}}^{\text{( t) , 3decays}}=\frac{1}{2}\frac{D_{\text{outer, 3}}^{\text{( t) , scale 1}}}{D_{\text{outer, 3}}^{\text{( t) , scale3}}}.$$ (9)

The time difference between the first and third single would be replaced in the equation for $D_{\text{outer, 2}}^{\text{( t) , 2delays}}$ by $\Delta t_{\max}=\max(\Delta t_{1,2},\Delta t_{1,3})$. The method is applicable to $\beta^{+}$- and $\beta^{+}\text{-}\gamma$-emitters.

#### 2.3.4 Rate estimation for two-decay random triples of $\beta^{+}\text{-}\gamma$-emitters identified with $V_{\text{outer}}$

The approach presented above is not suitable for two-decay random triple rates of $\beta^{+}\text{-}\gamma$-emitters, since second-order terms of $\epsilon_{1}\epsilon_{3}$, would also be included, with $\epsilon_{3}$ denoting the efficiency of detecting a true triple. Similar to the approach for the random double estimation, in this case, it is necessary to apply veto windows around the included single and the double (Figure 6F). Consequently, the veto interval is doubled and must be compensated for, e.g., by the approximately scaling factor $D_{\text{outer, 3}}^{\text{( t) , scale 1}}/D_{\text{outer, 3}}^{\text{( t) , scale 2}}$ (window scheme outer, 3, scale 1, shown in Figure 6D and window scheme outer, 3, scale 2, shown in Figure 6H). The three-decay triples contained within consist of unpaired singles only. Their rate is estimated using two delayed windows and veto windows around each single. This tripled veto interval size requires a correction factor given by $D_{\text{outer, 3}}^{\text{( t) , scale 1}}/D_{\text{outer, 3}}^{\text{( t) , scale3}}$ (window scheme outer, 3, scale 1, shown in Figure 6D and window scheme outer, 3, scale3, shown in Figure 6E). With these delayed coincidence rates, the random coincidence rate can be estimated by

$${\hat{R}}_{\mathrm{outer},b}^{\text{( t) , 2decays}}=\left(\frac{D_{\text{outer, 4}}^{\text{( t) , 1delay}}}{D_{\text{outer, 3}}^{\text{( t) , scale 2}}}-\frac{D_{\text{outer, 4}}^{\text{( t) , 2delays}}}{D_{\text{outer, 3}}^{\text{( t) , scale3}}}\right)D_{\text{outer, 3}}^{\text{( t) , scale 1}},$$ (10)

with b in the subscript indicating method b.

### 2.4 Simulation and measurement setup

**Figure 7:**
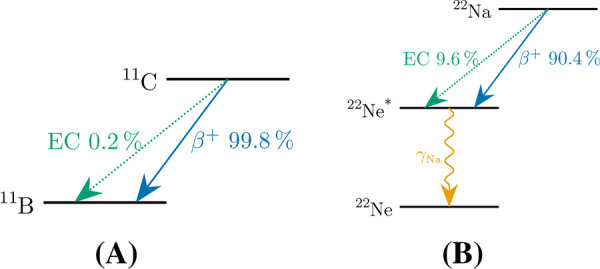
Decay schemes of the used $\beta^{+}$- and $\beta^{+}\text{-}\gamma$-emitters, **(A)** ^11^C, and **(B)** ^22^Na [Data extracted from [38]].

To validate the methods, MC simulations were performed using PeneloPET [23, 35, 36] and allowing access to the ground truth. The simulations were further experimentally validated (partly) using the recently developed BrainPET-7T [37]. Figure 7 shows the decay schemes of the $\beta^{+}$- and $\beta^{+}\text{-}\gamma$-emitters that were applied in the validation of our methods.

#### 2.4.1 BrainPET-7T and measurements

The BrainPET-7T is a high-performance multimodal imaging system that enables the simultaneous acquisition of ultrahigh-field MRI and PET for human neuroimaging [37]. With its high sensitivity (12% at the isocenter), the BrainPET insert offers excellent conditions for true triple detection of $\beta^{+}\text{-}\gamma$-emitters. The system consists of 120 scintillation detectors built with three staggered layers (24×24, 23×24, and 22×23) of lutetium oxyorthosilicate (LSO) crystals. The scintillation light is detected by an array of 12×12 digital silicon photomultipliers [39]. Further details of its properties can be found in Table 1.

**Table 1:** Parameters of the BrainPET7T and the simulated scanner design.

BrainPET-7T		Additional assumptions in the simulation	
Diameter ${\text{⌀}}_{\text{scanner}}$	40 cm	Scintillation pulse integration time	680 ns
Length l	25 cm	CTR	500 ps
Crystal width	2 mm	Dead time	10 ns
Crystal height layer 1/2/3	9/8/7mm	Energy resolution	14%
Reflector thickness	77.5μm		

**Figure 8:**
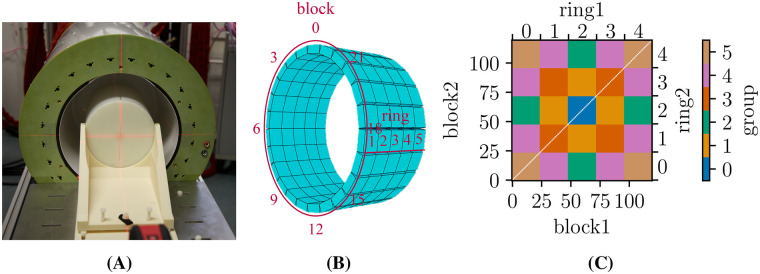
**(A)** Photo of the BrainPET-7T with a cylindrical phantom located at the isocenter. **(B)** Block/ring counting, **(C)** block groups of symmetrical locations, axial.

Using the BrainPET-7T, the following measurement was performed with the source positioned at the isocenter:

${\mathrm{MCS}}_{\text{C11}}$ Decay measurement of a cylindrical ${}^{11}C$-source (axial length 23.6cm, ⌀=14cm, PMMA, see Figure 8A) acquired for ≈1min each with activities in the range between 3 MBq and 100 MBq.

Each measurement was divided into five sets to estimate the uncertainty level of the results.

#### 2.4.2 Simulated systems and sources

The simulated scanner was configured as summarized in Table 1. For the simulations, the detector stack design of the BrainPET-7T scanner was slightly simplified, as the applied simulation was restricted to pixelated blocks with layers containing the same number of crystals (23×24). The final read-out hardware of 2×2 digital silicon photomultipliers was not simulated. However, these simplifications are not expected to affect the studied relationships between the trues and randoms.

The following sources were simulated:

${\mathrm{PS}}_{\text{ideal}}$ Ideal point source, ⌀=1mm.
${\mathrm{CS}}_{\text{ideal}}$ Ideal extended source, cylinder with ⌀=18cm and a height of 21cm.
${\mathrm{CS}}_{\text{real}}$ Geometry identical to ${\mathrm{CS}}_{\text{ideal}}$ with source surrounded by air and considering positron range, non-collinearity, and scattering/attenuation of the source, assuming water as the material.

For the simulations, all sources were positioned at the isocenter. The subscript *ideal* indicates the following properties of the corresponding source: absence of dead time^1^; the direct generation of annihilation photons, therefore neglecting positron range and non-collinearity; no photon interactions in the source; and a vacuum surrounding the source to further suppress scattering and attenuation. The simulated isotopes were ${}^{11}C$, as an example of a pure $\beta^{+}$-emitter, and ${}^{22}\mathrm{Na}$, as an example of a $\beta^{+}\text{-}\gamma$-emitter. The isotope symbols were added as superscript to the respective source names. A low-activity simulation was performed to determine the efficiencies $\epsilon_{1}$, $\epsilon_{2}$, and $\epsilon_{\geq 1}$ from the number of qualified singles and trues for the idealized cases, yielding $\epsilon_{1}=0.22$, $\epsilon_{2}=0.23$ and $\epsilon_{\geq 1}=0.45$ for ${\mathrm{PS}}_{\text{ideal}}$ and $\epsilon_{1}=0.4$, $\epsilon_{2}=0.14$ and $\epsilon_{\geq 1}=0.54$ for ${\mathrm{CS}}_{\text{ideal}}$.

Activities comparable to those in the measurements were applied. For the pure simulation studies, a range of activities from 10 to 100 MBq, in increments of 10 MBq, were sampled with an acquisition time of 0.1 s. To estimate the level of uncertainty in the simulated results, each simulation was repeated five times, reproducing the approach in the experimental measurements. To compensate for simplifications in the simulations when comparing simulated to measured rates, the simulated count rates were normalized to match the initial measured count rate.

### 2.5 Evaluation

For both ${\mathrm{CS}}_{\text{real}}$ and the experimental measurements, the singles were selected with an energy window from 400 to 650 keV for the annihilation γ-photons and 1,100–1,500 keV for the prompt $\gamma_{\text{Na}}$-photons. For triples of $\beta^{+}\text{-}\gamma$-emitters, combinations of two photons in the lower and one photon in the higher energy window were considered. For the ideal sources, the annihilation and $\gamma_{\text{\textbackslash{},prompt}}$-singles were additionally selected with a type flag in order to discard scatter and pile-up events. Using the scanner dimensions and coincidence time resolution (CTR), the coincidence time window was computed and set to $\tau =\frac{\sqrt{{\text{⌀}}_{\text{scanner}}^{2}+l^{2}}}{c}+2\cdot \text{CTR}=2.6\;\mathrm{ns}$, as shown in Table 1. This covers the maximum photon travel time along the diagonal through the BrainPET-7T, where c denotes the speed of light in a vacuum. In order to provide a comparison with large axial FOV PET scanner designs with an axial length of 1 m and an 82 cm ring diameter, the behavior of the coincidence rates was also investigated with the coincidence time window set to 4.7 ns [40]. For delayed coincidence windows, the delay was δ=5μs.

The count rates were studied at the scanner and scintillation block levels. For the latter, the coincident events were classified into block combinations corresponding to groups, as shown in Figure 8. Block combinations on the diagonal of the block combination matrix detect randoms of two singles on the same block. As these would cause additional complexity and would be omitted in the reconstruction in any case, they were discarded.

The error bars in all plots indicate the standard error across the five repeated simulations/measurements. We evaluated the behavior of trues, randoms, delayed coincidences, doubles, triples, and multiples. In the simulations, randoms and trues were classified by the decay ID of each single.

#### 2.5.1 Multiples

The relevance of accurately handling and correcting triples and higher-order multiples was evaluated to determine their impact on the overall accuracy of the results. To this end, the contributions of coincidences from order m for low activity (20 MBq) and high activity (100 MBq) were investigated for both coincidence time windows and for ${\mathrm{CS}}_{\text{ideal}}^{\text{C11}}$ with veto scheme $V_{\text{outer}}$. In addition, the ratio of pure triples to pure doubles, as identified with $V_{\text{outer}}$ for ${\mathrm{PS}}_{\text{ideal}}^{\text{C11}}$, ${\mathrm{CS}}_{\text{ideal}}^{\text{C11}}$, ${\mathrm{CS}}_{\text{real}}^{\text{C11}}$, and ${\mathrm{MCS}}_{\text{C11}}$ was also investigated.

#### 2.5.2 Veto schemes

The prompt rates across the veto schemes $V_{\text{between}}$ and $V_{\text{outer}}$ were compared for ${\mathrm{MCS}}_{\text{C11}}$ and ${\mathrm{CS}}_{\text{ideal}}^{\text{C11}}$ with varying activity levels. The fraction of trues detected as doubles (and triples) for veto schemes $V_{\text{outer}}$ and $V_{\text{inner}}$ per trues detected with $V_{\text{no}}$ (denoted as trues $V_{\text{no}}$) was also determined. This gives the maximum number of identifiable trues for the selected coincidence window. Furthermore, the proportion of ground truth trues in the prompts was determined for all veto schemes and ${\mathrm{CS}}_{\text{ideal}}^{\text{C11}}$, as well as ${\mathrm{CS}}_{\text{real}}^{\text{C11}}$.

#### 2.5.3 Rate estimation for random doubles

To evaluate the accuracy of the random estimation method, first the equations that led to the random estimators, Equations 2b, 3a, 3b, 3c, 4, and 5 were verified. Therefore, randoms were identified for ${\mathrm{PS}}_{\text{ideal}}$, the different veto schemes, and the corresponding delayed coincidences, using the schemes presented in Figure 3. The rates were then fitted to the following function:

$$R^{\text{2decays}}=aA^{2}\exp(\;-\;bA).$$ (11)

In Equation 11, A is the source activity and a and b (b=0 for $V_{\text{no}}$) are free fit parameters depending on scanner characteristics, such as detection efficiency. In addition, the randoms were estimated for ${\mathrm{CS}}_{\text{real}}$ according to the described methods. They were then compared to the ground truth randoms.

#### 2.5.4 Rate estimation for random triples of a pure $\beta^{+}$-emitter

An examination of ${\mathrm{CS}}_{\text{real}}^{\text{C11}}$ with an activity of 50 MBq was conducted with τ=4.7ns and $V_{\text{outer}}$ to assess the contributions of different triple types for different window schemes and the contributions were replicated for ${\mathrm{CS}}_{\text{ideal}}^{\text{C11}}$ and τ=8ns. To validate Equations 6, 8, ${\mathrm{CS}}_{\text{ideal}}^{\text{C11}}$ was simulated as a function of the activity and the two-decay random rates were fitted according to Equation 11 and the three-decay random rates were fitted to

$$R^{\text{3decays}}=aA^{3}\exp(\;\;-\;bA)$$ (12)

for τ=8ns. The extended source was chosen because interval II (Figure 4C) is negligible for PS${}_{\text{ideal}}$. In addition, the accuracy of the prediction of random rate estimations for two- and three-decay triples was also evaluated for different ${\mathrm{CS}}_{\text{real}}^{\text{C11}}$ activities.

#### 2.5.5 Rate estimation for random triples of a $\beta^{+}\text{-}\gamma$-emitter

For ${\mathrm{CS}}_{\text{real}}^{\text{Na22}}$ and different activities, the contributions of triples were divided depending on the number and order of singles from different decays and the number of prompts identified with $V_{\text{no}}$, $V_{\text{between}}$, $V_{\text{inner}}$, and $V_{\text{outer}}$ were studied. The proportion of contained two- and three-decay random triples was determined for $V_{\text{no}}$ and $V_{\text{outer}}$ (using Equations 9, 10) and the accuracy of the prediction of their rates was evaluated.

## 3 Results

### 3.1 Contribution of multiples

**Figure 9:**
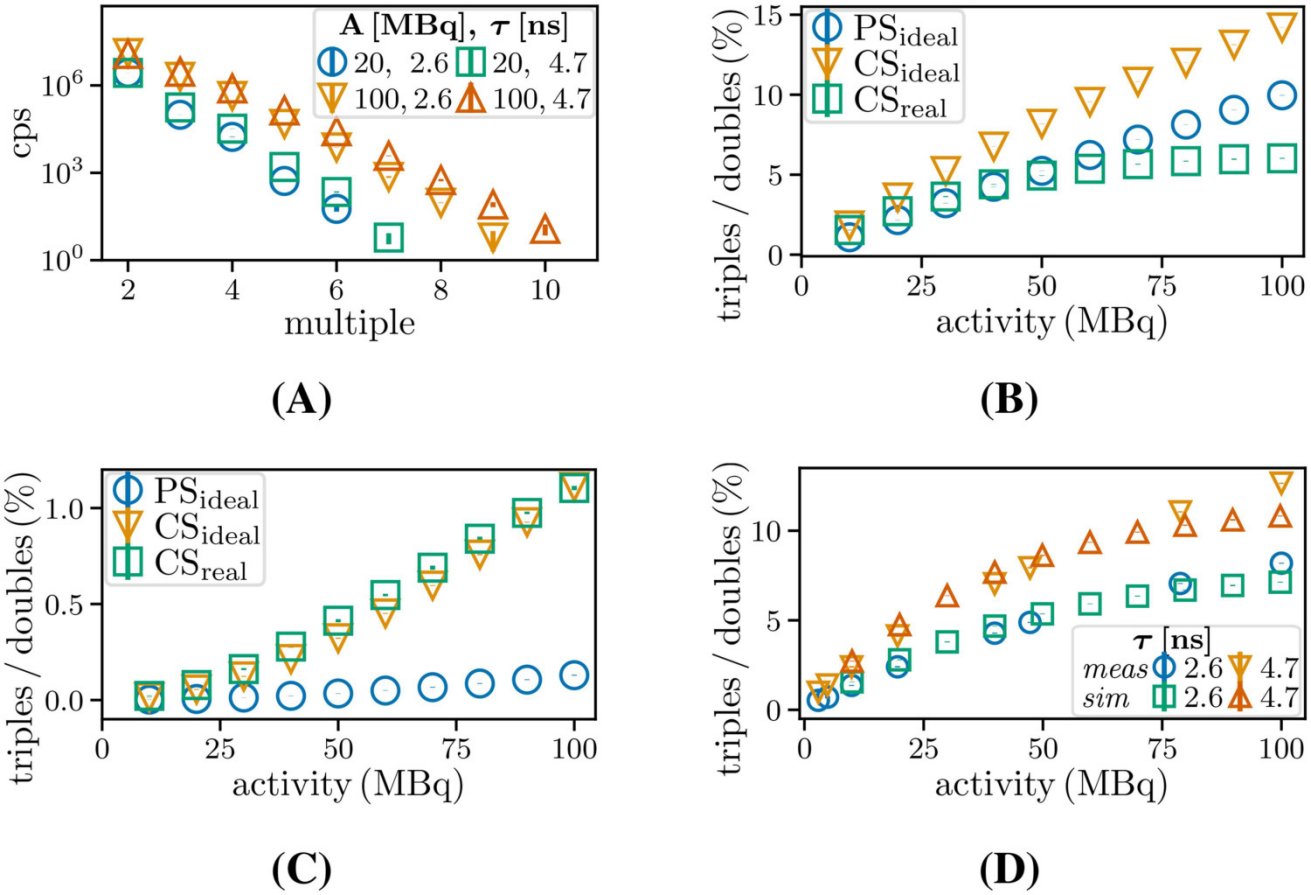
**(A)** Contribution of different multiples for two activities and coincidence time windows for ${\mathrm{CS}}_{\text{ideal}}^{\text{C11}}$. Ratio of prompt triples to doubles for ${\mathrm{PS}}_{\text{ideal}}^{\text{C11}}$, ${\mathrm{CS}}_{\text{ideal}}^{\text{C11}}$, and ${\mathrm{CS}}_{\text{real}}^{\text{C11}}$ for **(B)** two- and **(C)** three-decay triples and for ${\mathrm{MCS}}_{\text{C11}}$ and ${\mathrm{CS}}_{\text{real}}^{\text{C11}}$ **(D)** ${\mathrm{MCS}}_{\text{C11}}$.

Figure 9 shows the exponential decline in the detections of prompt multiples for ${\mathrm{CS}}_{\text{ideal}}^{\text{C11}}$ and $V_{\text{outer}}$ obtained for the two specified coincidence time windows and activities. The rate of triples for 100 MBq is comparable to the rate of doubles for 20 MBq. For odd orders of multiples, the minimum number of decays required is higher by one when compared to the preceding order of multiples. In contrast, for even orders of multiples, the minimum number of decays remains the same, scaling with the ratio in order to detect a paired single instead of an unpaired one. This results in count rate differences for subsequent orders of multiples that slightly alternate in magnitude.

Figures 9B, C present the ratio of prompt triples to doubles for two-decay and three-decay triples in $V_{\text{outer}}$. For all sources, the increasing ratio reaches 5% at 50 MBq, reaching higher values of 10% for ${\mathrm{PS}}_{\text{ideal}}^{\text{C11}}$ and 14% for ${\mathrm{CS}}_{\text{ideal}}^{\text{C11}}$ at 100 MBq. For three decays, the ratio reaches 1% at 90 MBq for both ${\mathrm{CS}}_{\text{real}}^{\text{C11}}$ and ${\mathrm{CS}}_{\text{ideal}}^{\text{C11}}$, while ${\mathrm{PS}}_{\text{ideal}}^{\text{C11}}$ exhibits an even lower value due to the reduced probability of detecting singles as unpaired singles. Figure 9D shows the triples to doubles ratio for ${\mathrm{MCS}}_{\text{C11}}$ and ${\mathrm{CS}}_{\text{real}}^{\text{C11}}$ for two coincidence window sizes. The simulations and experimental measurements are in good agreement, and the ratio is increased by a factor of ≈1.8, corresponding to the ratio of the window lengths.

### 3.2 Impact of different veto schemes

**Figure 10:**
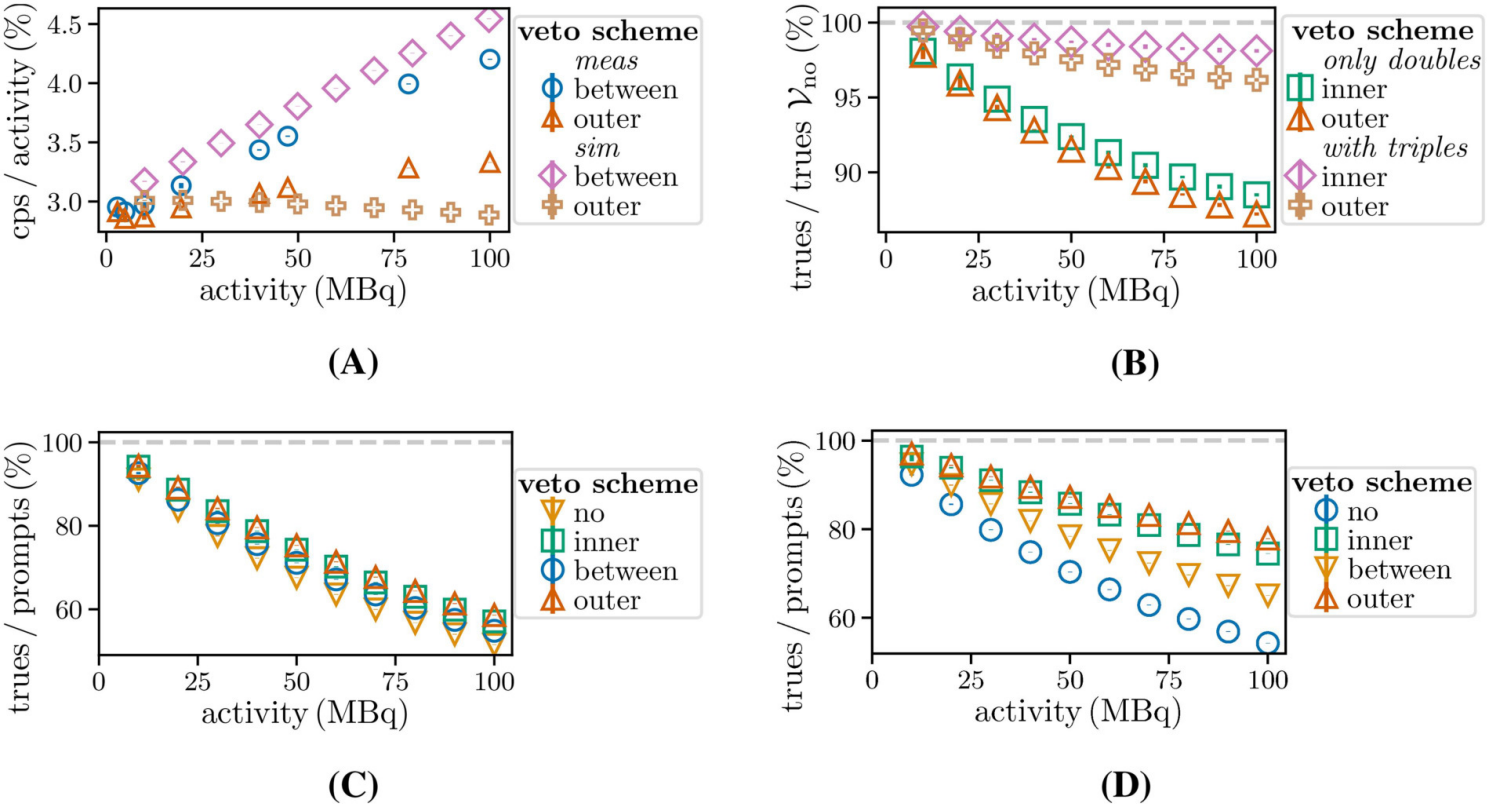
**(A)** Prompt double rates per activity for ${\mathrm{MCS}}_{\text{C11}}$ (meas) and ${\mathrm{CS}}_{\text{ideal}}^{\text{C11}}$ (sim, scaled by 4.5). **(B)** Fraction of accepted trues, and the ratio between double trues and prompts for **(D)** ${\mathrm{CS}}_{\text{real}}$ and **(C)** ${\mathrm{CS}}_{\text{ideal}}^{\text{C11}}$.

Figure 10A compares the prompt count rates per activity for $V_{\text{outer}}$ and $V_{\text{between}}$ for ${\mathrm{CS}}_{\text{ideal}}^{\text{C11}}$ (scaled by a factor of 4.5) and ${\mathrm{MCS}}_{\text{C11}}$. The ratios for $V_{\text{between}}$ increase with the activity. In contrast, the prompt count rates are necessarily lower for $V_{\text{outer}}$ due to the requirement of adjacent single-free intervals. The ratio shows a slower increase with the activity for ${\mathrm{MCS}}_{\text{C11}}$. However, the rise in randoms with higher activity is offset by the reduction in single free intervals for ${\mathrm{CS}}_{\text{ideal}}^{\text{C11}}$ with higher single detection efficiency. Figure 10B shows a comparable decline in the detected fraction of true doubles for $V_{\text{inner}}$ and $V_{\text{outer}}$. At approximately 30 MBq, 5% of trues are rejected as pure doubles. Most of the excluded true doubles are identified as being contained in triples. Figure 10C illustrates that at 10 MBq the ratio of trues to prompts is (91.08±0.25)% for $V_{\text{no}}$ and 3% higher for $V_{\text{outer}}$. Notably, this difference increases to 7%. However, even for $V_{\text{outer}}$, the random fraction reaches (42.48±0.04)%, highlighting the need for adequate random estimation. The differences are enhanced for ${\mathrm{CS}}_{\text{ideal}}^{\text{C11}}$ (Figure 10D) due to the higher detection efficiency $\epsilon_{2}$.

### 3.3 Rate estimation for random doubles

**Figure 11:**
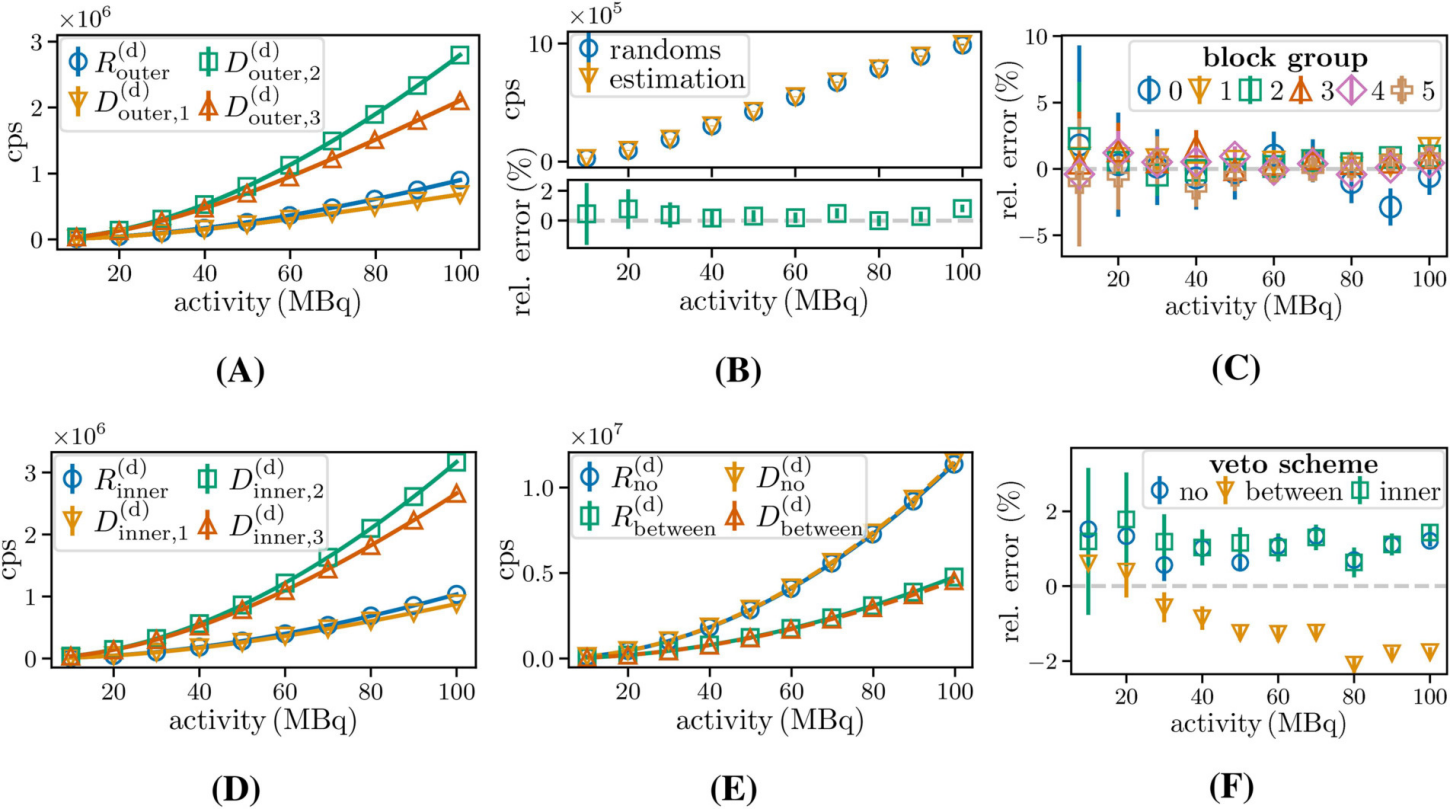
Upper row: **(A)** random rate estimation of doubles for $V_{\text{outer}}$ and for ${\mathrm{CS}}_{\text{real}}^{\text{C11}}$. **(B)** Rates with different window schemes, estimation with Equation 3d compared to true randoms on the scanner level, and **(C)** deviation for different block groups dependent on the activity. Lower row: Random rate estimation for $V_{\text{no}}$, $V_{\text{between}}$, and $V_{\text{inner}}$. Random rate and rates with fit for **(D)** $V_{\text{inner}}$, **(E)** $V_{\text{no}}$, and $V_{\text{between}}$ and **(F)** comparison between random rate and estimation.

Figure 11A shows the count rate fits for randoms and the associated delayed window schemes for $V_{\text{outer}}$. The corresponding best-fit parameters are listed in Table 2. The reduced $\chi^{2}$ value, $\chi_{\text{red}}^{2}$, is obtained by dividing the $\chi^{2}$ statistic by its degrees of freedom (8, 10 data points and two fit parameters). The values of the best-fit parameters are in agreement with the parameters in Equations 2b, 3a, 3b, 3c (with a maximum deviation of 2%).

**Table 2:** Best-fit parameters for random and delayed double and triple coincidences using Equations 11, 12.

Parameters for random and delayed double rates for different veto schemes shown in Figure 11.							
	a[ps]	b[ns]	$\chi_{\text{red}}$		a[ps]	b[ns]	$\chi_{\text{red}}$
$R_{\text{outer}}^{\text{( d) }}$	120.87(25)	2.909 (28)	0.38	$R_{\text{inner}}^{\text{( d) }}$	123.07 (26)	1.740 (28)	0.48
$D_{1}^{\text{( d) }}$	120.4 (4)	5.65 (5)	1.29	$D_{1b}^{\text{( d) }}$	122.8 (3)	3.33 (4)	1.88
$D_{2}^{\text{( d) }}$	372.8 (6)	2.829 (20)	0.71	$D_{2b}^{\text{( d) }}$	374.2 (6)	1.661 (21)	0.73
$D_{3}^{\text{( d) }}$	372.7 (5)	5.680 (17)	0.37	$D_{3b}^{\text{( d) }}$	373.4 (7)	3.382 (24)	2.58

$R_{\text{no}}^{\text{( d) }}$	1142.8 (3)		2.20	$R_{\text{between}}^{\text{( d) }}$	507.5 (3)	0.591 (8)	0.55
$D_{\text{no}}^{\text{( d) }}$	1154.2 (6)		3.36	$D_{\text{between}}^{\text{( d) }}$	510.7 (7)	1.109 (17)	2.91

Parameters for triple random rates shown in Figure 12A.				
		a [ps]	b [ns]	$\chi_{\text{red}}$
	I	236.5 (3)	6.294 (19)	0.61
2 decays	II	13.62 (10)	5.22 (9)	0.89
	III	236.1 (4)	6.274 (18)	0.55
		a [${\mathrm{ns}}^{2}]$	b [ns]	$\chi_{\text{red}}$
3 decays		0.691 (26)	6.442 (15)	0.79

Parameters for delayed triple rates shown in Figure 12B.				
		a[ps]	b[ns]	$\chi_{\text{red}}$
$D_{\text{outer, 4}}^{\text{( t) , 1delay}}$	2 decays	11.600 (15)	3.388 (14)	2.30
		a [${\mathrm{ns}}^{2}$]	b [ns]	$\chi_{\text{red}}$
$D_{\text{outer, 4}}^{\text{( t) , 1delay}}$	3 decays	0.01282 (8)	2.97 (7)	0.14
$D_{\text{outer, 4}}^{\text{( t) , 2delays}}$	3 decays	0.11602 (21)	3.352 (26)	0.55

Figure 11B shows the random rates extracted from the simulations and the estimated rates (via delayed randoms) and the deviations between them. Figure 11C illustrates the deviations for the different block groups. A maximum deviation of (0.82±0.24)% at the scanner level and (2.9±1.4)% at the block level is obtained. The results indicate that the proposed estimation method provides an estimator for the random contributions to the prompt count rates, achieving a relative error of <1% at the scanner level. Figures 11D, E show the determined random and coincidence rates, with fits to the model in Equation 11 (with b=0 for $V_{\text{no}}$) for the three other veto schemes. The obtained fit values deviate at a maximum of 4% from the assumed values for detection efficiencies and time distances in the models. An additional study of the expected time difference ⟨Δt⟩ revealed that the activity and veto scheme-dependent relative discrepancy from 0.5τ was in a range of ±3%. The deviation between random estimation and randoms in the prompt window shown in Figure 11F reaches a maximum of 2% for $V_{\text{between}}$. Estimates for $V_{\text{inner}}$ have a ≈1% deviation. The same is found for $V_{\text{no}}$, where the same combinations are considered in the prompt windows, and the bias is assumed to be due to dead time, which reduces the detected number of singles in the prompt window. The results show that all the estimators, including those based on ratios, provide an accurate random estimation.

### 3.4 Rate estimation for random triples of a pure $\beta^{+}-$emitter

**Figure 12:**
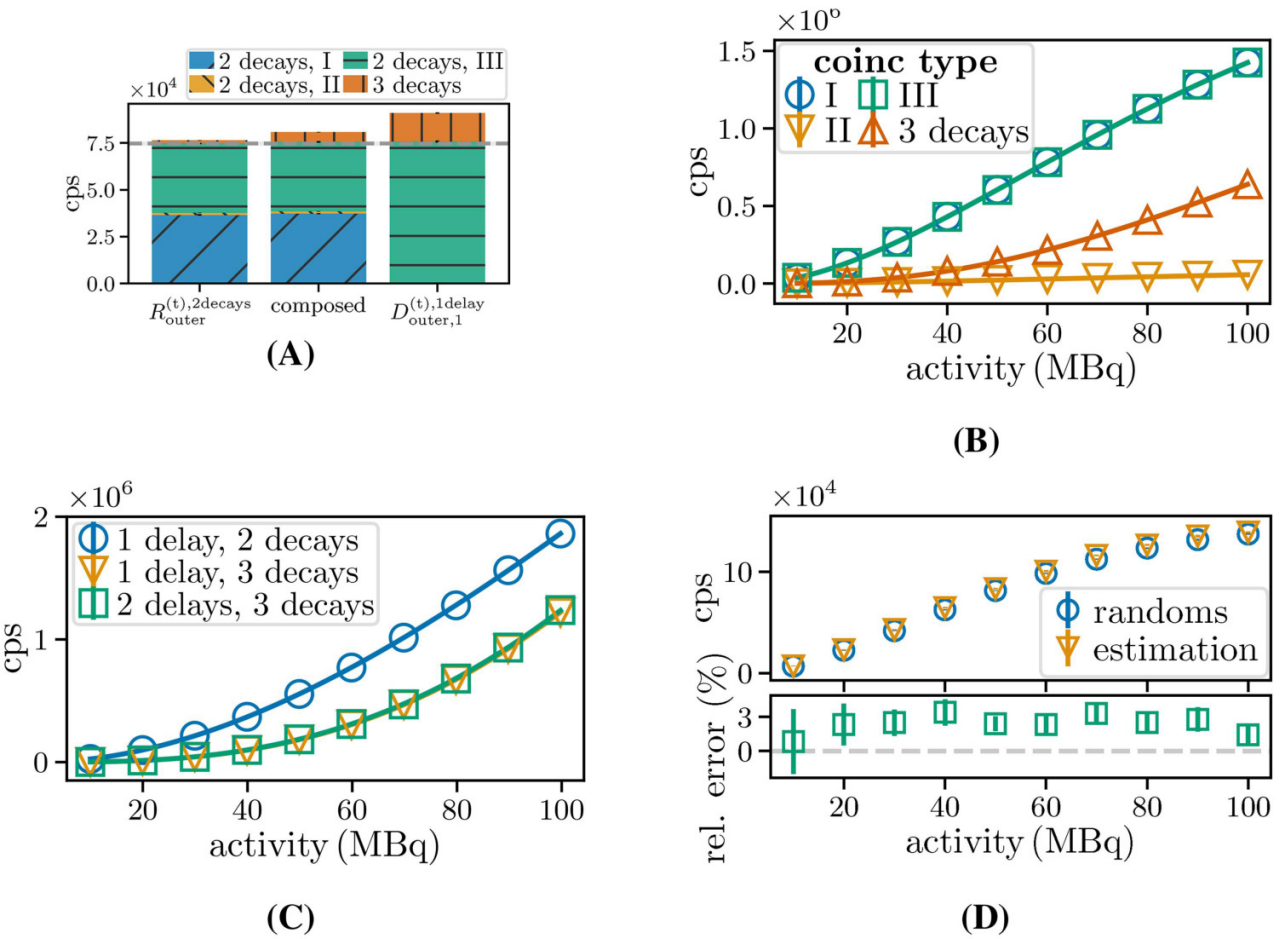
**(A)** Histogram of random triples, composed of counts of delayed triples determined by different window schemes (for identifying the contributions of the intervals explained in Figure 5) and $D_{\text{outer, 1}}^{\text{( t) , 1delay}}$. The pattern indicates the triple type, and the horizontal line marks the counts of the two-decay randoms. **(B)** Random triple and **(C)** delayed coincidences fits for ${\mathrm{CS}}_{\text{ideal}}^{\text{C11}}$ and **(D)** estimation for two-decay triple rates for ${\mathrm{CS}}_{\text{real}}^{\text{C11}}$.

Figure 12A shows stacks of the rates of different coincidence types for prompts identified by $V_{\text{outer}}$ for ${\mathrm{CS}}_{\text{ideal}}^{\text{C11}}$ simulations, next to the contributions divided into the parts shown in Figure 5 and for $D_{\text{outer, 1}}^{\text{( t) , 1delay}}$. It can be seen that the same level of two-decay triples (grey line) is reached, while the rate of three-decay triples is higher for $D_{\text{outer, 1}}^{\text{( t) , 1delay}}$.

Figure 12B shows the count rate fits of simulated and estimated randoms for the different coincidence types. The best-fit parameters obtained (Table 2) are in good agreement with the parameters in Equation 7.

The fits for the delayed rates $D_{\text{outer, 1}}^{\text{( t) , 2delays}}$ and $D_{\text{outer, 1}}^{\text{( t) , 1delay}}$ for ${\mathrm{CS}}_{\text{ideal}}^{\text{C11}}$, along with their corresponding count rates, are shown in Figure 12B and the best-fit parameters, which lead to a maximum deviation of 3% from the expected value, are listed in Table 2. Figure 12C compares the random rate and its estimator. It can be seen that, with an average percentage difference of (2.4±0.4)% and a maximum percentage difference (3.3±0.7)%, our estimation method also achieves high accuracy for random triple estimation.

### 3.5 Rate estimation for random triples of a $\beta^{+}\text{-}\gamma$-emitter

**Figure 13:**
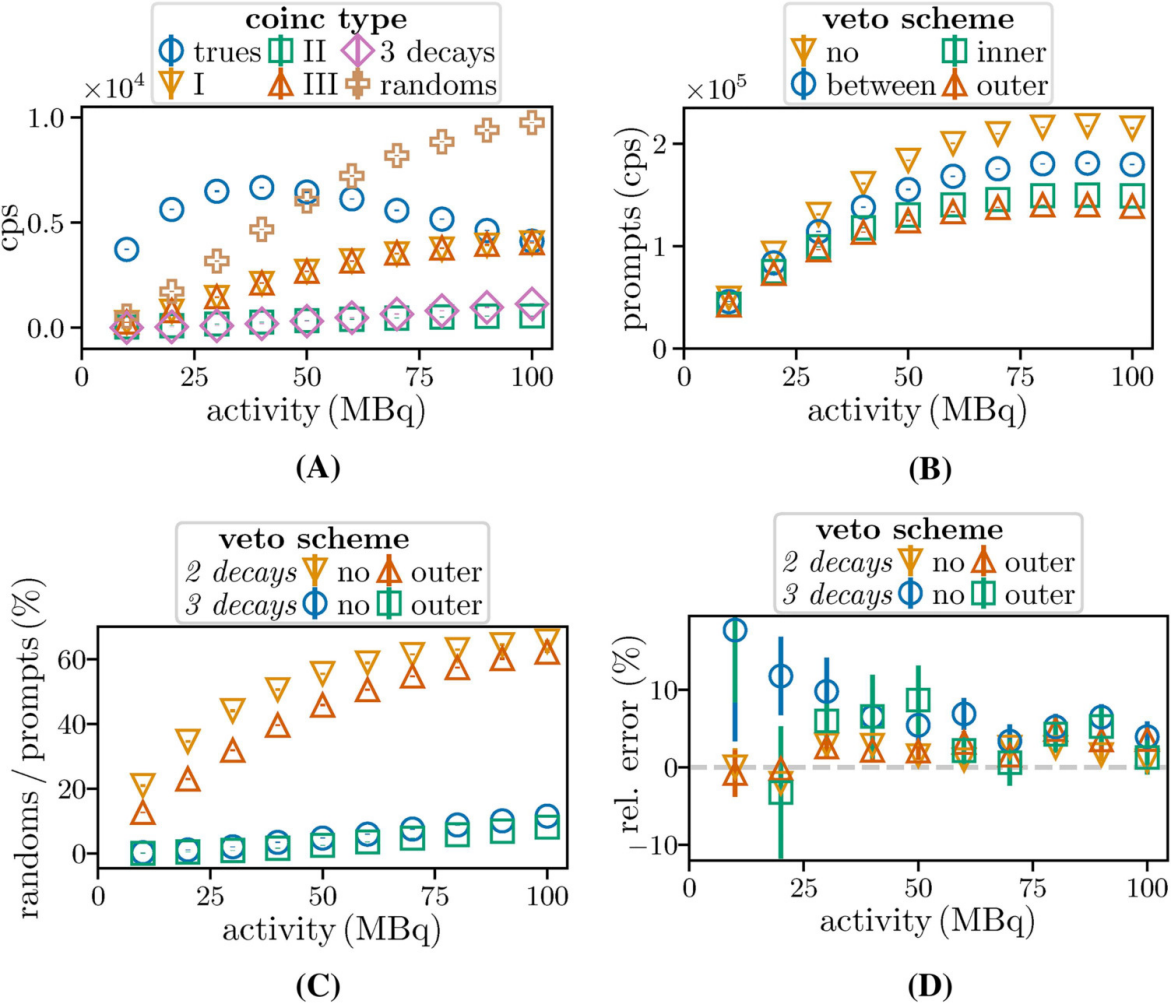
Triple count rates for ${\mathrm{ES}}_{\text{real}}^{\text{Na22}}$ **(A)** divided into coincidence types, **(B)** prompts for different veto schemes, **(C)** the proportion of contained randoms divided into two- and three-decay randoms, and **(D)** the relative deviation of their estimators. The value of the first deviation of (60±60)% for ${\hat{R}}_{\mathrm{outer}}^{\text{( t) , 3decays}}$ is discarded for better representation of all other values.

Figure 13 shows the contribution to randoms and estimations for ${\mathrm{CS}}_{\text{real}}^{\text{Na22}}$. In Figure 13A, it can be seen that the randoms (sum of all random types) already exceed the trues at 60 MBq, and that the contributions I and III both reach the same values as the trues at 100 MBq. Figure 13B shows the prompt rates obtained by the simulations for the four veto schemes. The ratio of prompts identified with $V_{\text{no}}$ and $V_{\text{outer}}$ is approximately 1.5 the highest investigated activity. Figure 13C depicts the proportion of randoms in prompts separated for two- and three-decay triples for $V_{\text{no}}$ and $V_{\text{outer}}$. The maximum deviation for two-decay triples is 12% at 30 MBq. At 100 MBq, 3% fewer randoms per prompts for both random types are identified with $V_{\text{outer}}$. Thereby, 16% of trues identified with $V_{\text{no}}$ are excluded, along with exclusion of 39% and 45% of two- and three-decay triples, respectively. The relative percentage deviation between the random rate and estimated rate for two- and three-decay triples according to Equations 9, 10 with the corresponding deviations for $V_{\text{no}}$ at different activities, are shown in Figure 13D. For the case of two decays, the deviation between the actual randoms and the random estimation ranges from −1% to 5% for $V_{\text{outer}}$ and −2% to 3% for $V_{\text{no}}$. The maximum deviations for the case of three decays range from (60±60)% and 12%, respectively, and the average deviations are 4% and 6%.

## 4 Discussion

This study suggests that the inclusion of random triples should be considered in order to achieve accurate quantitative reconstruction. Notably, the results show that at 50 MBq random triples can account for 5% of the detected doubles in the simulated scanner geometry for $V_{\text{outer}}$ using both a point source and an extended source. However, the alternative solution of suppressing random triples by using veto schemes would compromise detection sensitivity, depending on the strictness of the exclusion. For example, in the case of the sources studied at 30 MBq, this would result in the rejection of 5%–10% of trues compared to $V_{\text{no}}$. In our proposed approach, doubles and triples are identified separately, and therefore, triples do not contribute to doubles. The trues rejected with this method are mainly recovered in the identified triples (as double counting was not prohibited for $V_{\text{inner}}$, the sum of trues in doubles and triples may be slightly higher than the actual number of contributing trues). While with $V_{\text{inner}}$, all coincidences would be accepted by adding up a sufficiently high order of multiples, with some singles being part of different coincidences, $V_{\text{outer}}$ avoids repeated consideration, but cannot recover all trues, even after increasing the identified multiple order. Conversely, the application of the veto windows leads to a significant reduction in randoms, particularly for source distributions with a high acceptance of randoms containing paired singles.

Discrepancies between the simulated and measured results can be partly explained by several factors. First, the simulated design was slightly adjusted to include layers with the same number of crystals, and the detector and source housing was omitted. In addition, a slightly lower energy resolution and higher time resolution were employed, along with an assumption of a higher dead time compared to the experimental system (assumed parameters before the system became operational). Furthermore, this value was set on a block level in the simulation, while it is on a silicon photomultiplier (SiPM) level in the experimental system.

Our models can account for the various contributions to random and delayed coincidences for both doubles and triples. Notably, our results show a lower relative deviation than that reported in [15] for the investigated delayed window methods and simulation setups. The observed overestimation can be potentially explained by dead time, which is expected to affect the detected singles in the prompt window more than in the delayed window. This effect is even stronger for randoms containing paired singles. In our study, random rates were found to be reduced for correlated singles, which, however, still contribute to dead time. The observation that dead time affects count rates in the prompt and delayed coincidence window differently is supported by the results presented in [15], where no differences were reported when placing the source outside of the scanner FOV. However, owing to its complex behavior, the interaction between dead time and count rates in different coincidence windows should be studied in an additional dedicated simulation study, as it is beyond the scope of this work.

The random double estimation method for $V_{\text{outer}}$ results in an estimator with a deviation of <1% at the scanner level for all activities studied by simulation. The high standard error at the block level reveals that variance reduction methods, as described in [17], are required when considering lines of responses between crystals. We expect the aforementioned variance reduction to be compatible with the investigated methods and anticipate that it could be combined with an extension of the delayed window techniques [41]. For the other veto schemes, a small relative deviation of ≈1% was found.

The results show that our method for the estimation of two-decay random triple rates of $\beta^{+}$-emitters provides an accurate estimator with a deviation <4% for all cases and activities studied by MC simulations. A further important finding of our study is that a second delayed window for exclusively identifying three-decay triples is required for the accurate estimation of random triple rates. This is also necessary for the correct estimation of two-decay random triple rates. The window intervals we obtained for the estimation of random triples are in agreement with the windows in [34], starting from the first annihilation photon without veto windows, where the method was used for random corrected image reconstruction applied to positron lifetime imaging. However, the accuracy of the delayed window method for triple randoms was not evaluated in their work. With this approach, three-decay random triples are overestimated by ≈5%; however, it should be noted that the statistical uncertainty of this measure is rather high. Two-decay random triples of $\beta^{+}\text{-}\gamma$-emitters, which already exceed the number of trues at 30 MBq, are overestimated by <3% for $V_{\text{no}}$ and <5% for $V_{\text{outer}}$. One factor in the observed inconsistency is the oversimplification of the difference between the first and second (third) single $\left\langle \Delta t_{1,2(3)}\right\rangle$ for two-decay and three-decay triples contributing to the scaling factors for the veto window size. The exact consideration would require a more complex composition of rates identified with different coincidence identification schemes. Furthermore, scatter and multiples of orders higher than three need to be considered to obtain a more accurate estimator.

Several research groups have explored potential ways of adequately integrating triples into PET image reconstruction. Andreyev and Celler [2], Pratt et al. [4], and Pfaehler et al. [42] present options to exploit the triples from $\beta^{+}\text{-}\gamma$-emitters to enable the separation of images from a $\beta^{+}\text{-}\gamma$-emitter and a $\beta^{+}$-emitter in dual-tracer PET. In Pfaehler et al. [42], an extension to the state-of-the-art maximum likelihood expectation maximization (ML-EM) reconstruction method was proposed that uses an additional gamma photon to separate the contributions from the $\beta^{+}-$ and $\beta^{+}\text{-}\gamma$-emitter during reconstruction. However, their study did not include random coincidences or scattered events due to a lack of estimation methods. Instead, Pfaehler et al. [42] used ground truth information from MC simulations to distinguish between doubles and triples. The triple random estimation methods developed here can be used to extend Pfaehler et al.’s method by incorporating the corresponding random estimators into the forward models for $\beta^{+}-$ and $\beta^{+}\text{-}\gamma$ image reconstruction, in the same way that double random coincidences were considered in the standard PET forward model. The most suitable veto scheme for this scenario still needs to be determined.

For $\beta^{+}$-emitters, pure doubles identified with $V_{\text{outer}}$ and identified triples could be treated separately in the reconstruction. Gillam et al. [25] suggest that their V-projection recovery method for inter-crystal scatter triples can also be used for adequate consideration of two-decay triples. Lage et al. [26] also propose a proportional method that weights inter-detector scatter triples (extended by Lee et al. [43] for inter-crystal scatters) by the relative proportion of doubles in the potential lines of response. Whether an improvement in quantitation accuracy can actually be achieved with these implementations requires further evaluation in a follow-up study.

Positronium lifetime and the possible differences in detection due to the higher energy of the $\gamma_{\text{\textbackslash{},prompt}}$ can impact the temporal structure of a triple. Notably, positronium lifetime was not taken into account in the simulations conducted in this study. Furthermore, in the random rate estimations, we did not split the triples according to the $\gamma_{\text{\textbackslash{},prompt}}$-single-position to account for the strongly differing frequencies of the positions. It should also be mentioned that for materials with a longer positronium-lifetime, the coincidence window needs to be enlarged in order to lose fewer true triples, which would, however, increase the fraction of randoms. Widening the coincidence time window by 2 ns to account for the typical ortho-positronium lifetime in the human body, which is between 1.8 and 2.5 ns [6], would result in the random level becoming comparable to the true level at approximately 20 MBq. A compromise must be found between enlarging the coincidence window to accept more trues and the increase in randoms that this causes. It should also be investigated whether extending the veto schemes to quadruples and higher-order multiples, incorporating them with different weights as suggested for $\beta^{+}$-emitter doubles and triples, can improve the quantitation accuracy.

## 5 Conclusion

This study examined the relevance of considering random triples in coincidence processing for $\beta^{+}$-emitters and the impact of different treatments of multiples on doubles identification, including the option to separate doubles and triples. Based on the delayed window technique, we propose corresponding methods for estimating the double and random triple rates, achieving relative deviations of <5% at the scanner level in simulations. Furthermore, we investigated randoms of $\beta^{+}\text{-}\gamma$-emitters using various identification schemes for multiples and introduced random estimation methods that yielded relative deviations of <5% in simulation studies. In future work, variance reduction will be implemented in the random estimations, and the impact of the methods on reconstructed images from standard measurements and dual-tracer PET will be investigated.

## Data Availability

The raw data supporting the conclusions of this article will be made available by the authors, without undue reservation.

## Footnotes

^1^We opted for a value of 100fs rather than 0 s to prevent artifacts in the analyzed data that could potentially be caused by the simulation. In addition, setting the integration time to 1 ns also resulted in a short dead time.

## Glossary

CTR: coincidence time resolution
MC simulation: Monte Carlo simulation
single: detected individual scintillation event assigned to one γ-photon
prompt: coincidence with at least two singles within a prompt coincidence time window
true: prompt containing only singles originating from the same decay
random: prompt containing at least one uncorrelated single (i.e., from a different decay)
delayed coincidences: coincidence with at least one event within a delayed coincidence window
double: two singles forming a coincidence
triple: three singles forming a coincidence
multiple: coincidence containing m singles with order m>2 (m>3 for triples)
two-decay triple: triple containing a true double and a single originating from a different decay
three-decay triple: triple containing singles originating from three different decays
paired single: single for which a single associated with the other photon from the annihilation pair was detected
unpaired single: single for which its other photon from the annihilation pair was not detected
veto schemes: time intervals which are required to be free of singles created around single’s timestamps

## References

[1] Cherry SR Sorenson JA Phelps ME, Physics in Nuclear Medicine. Philadelphia: W.B. Saunders (2012).

[2] Andreyev A Celler A. Dual-isotope PET using positron-gamma emitters. Phys Med Biol. (2011) 56:4539–56. 10.1088/0031-9155/56/14/020 21725143

[3] Conti M Eriksson L. Physics of pure and non-pure positron emitters for PET: a review and a discussion. EJNMMI Phys. (2016) 3:8. 10.1186/s40658-016-0144-5 27271304 PMC4894854

[4] Pratt EC Lopez-Montes A Volpe A Crowley MJ Carter LM Mittal V, et al. Simultaneous quantitative imaging of two PET radiotracers via the detection of positron–electron annihilation and prompt gamma emissions. Nat Biomed Eng. (2023) 7:1028–39. 10.1038/s41551-023-01060-y 37400715 PMC10810307

[5] Harpen MD. Positronium: review of symmetry, conserved quantities and decay for the radiological physicist. Med Phys. (2004) 31:57–61. 10.1118/1.1630494 14761021

[6] Moskal P Kisielewska D Curceanu C Czerwiński E Dulski K Gajos A, et al. Feasibility study of the positronium imaging with the J-PET tomograph. Phys Med Biol. (2019) 64:055017. 10.1088/1361-6560/aafe20 30641509

[7] Moskal P Jasińska B Stȩpień EŁ Bass SD. Positronium in medicine and biology. Nat Rev Phys. (2019) 1:527–9. 10.1038/s42254-019-0078-7

[8] Takyu S Ikeda H Wakizaka H Nishikido F Matsumoto K Tashima H, et al. Positron annihilation lifetime measurement with TOF-PET detectors: feasibility of Iodine-124 use. Appl Phys Express. (2023) 16:116001. 10.35848/1882-0786/ad047c

[9] Steinberger WM Mercolli L Breuer J Sari H Parzych S Niedzwiecki S, et al. Positronium lifetime validation measurements using a long-axial field-of-view positron emission tomography scanner. EJNMMI Phys. (2024) 11:76. 10.1186/s40658-024-00678-4 39210079 PMC11362402

[10] Evans RD. The Atomic Nucleus. New York: McGraw-Hill (1955). p. 791–3.

[11] Knoll GF. Radiation Detection and Measurement. 4th ed. New York: John Wiley & Sons (2010).

[12] Brasse D Kinahan PE Lartizien C Comtat C Casey M Michel C. Correction methods for random coincidences in fully 3D whole-body PET: impact on data and image quality. J Nucl Med. (2005) 46:859–67. 15872361

[13] Tetrault M-A Oliver JF Bergeron M Lecomte R Fontaine R. Real time coincidence detection engine for high count rate timestamp based PET. IEEE Trans Nucl Sci. (2010) 57:117–24. 10.1109/TNS.2009.2038055

[14] Oliver JF Rafecas M. Improving the singles rate method for modeling accidental coincidences in high-resolution PET. Phys Med Biol. (2010) 55:6951–71. 10.1088/0031-9155/55/22/022 21048288

[15] Oliver J.F. Rafecas M. Chen C-T. Modelling random coincidences in positron emission tomography by using singles and prompts: a comparison study. PLoS One. (2016) 11:e0162096. 10.1371/journal.pone.0162096 27603143 PMC5014417

[16] Badawi RD Miller MP Bailey DL Marsden PK. Randoms variance reduction in 3D PET. Phys Med Biol. (1999) 44:941–54. 10.1088/0031-9155/44/4/010 10232807

[17] Byars L Sibomana M Burbar Z Jones J Panin V Barker W, et al. Variance reduction on randoms from delayed coincidence histograms for the HRRT. In: *2005 IEEE Nucl Sci Symp Conf Rec*. IEEE (2005). Vol. 5. p. 2622–6.

[18] Strydhorst J Buvat I. Redesign of the GATE PET coincidence sorter. Phys Med Biol. (2016) 61:N522–31. 10.1088/0031-9155/61/18/N522 27589353

[19] Issa ASM Scheins J Tellmann L Lopez-Montes A Herraiz JL Brambilla CR, et al. A detector block-pairwise dead time correction method for improved quantitation with a dedicated BrainPET scanner. Phys Med Biol. (2022) 67:235004. 10.1088/1361-6560/aca1f3 36356317

[20] Oliver JF Torres-Espallardo I Fontaine R Ziegler S Rafecas M. Comparison of coincidence identification techniques for high resolution PET. In: *2008 IEEE Nuclear Science Symposium Conference Record*. Dresden: IEEE (2008). p. 4732–5.

[21] Pál L Pázsit I. On some problems in the counting statistics of nuclear particles: investigation of the dead time problems. Nucl Instrum Methods Phys Res Sect A. (2012) 693:26–50. 10.1016/j.nima.2012.07.036

[22] Germano G Hoffman EJ. Investigation of count rate and deadtime characteristics of a high resolution PET system. J Comput Assist Tomogr. (1988) 12:836–46. 10.1097/00004728-198809010-00021 3262635

[23] Cal-González J Lage E Herranz E Vicente E Udias JM Moore SC, et al. Simulation of triple coincidences in PET. Phys Med Biol. (2015) 60:117–36. 10.1088/0031-9155/60/1/117 25479147

[24] Leung EK Judenhofer MS Cherry SR Badawi RD. Performance assessment of a software-based coincidence processor for the EXPLORER total-body PET scanner. Phys Med Biol. (2018) 63:18NT01. 10.1088/1361-6560/aadd3c 30152793 PMC6354919

[25] Gillam JE Solevi P Oliver JF Casella C Heller M Joram C, et al. Sensitivity recovery for the AX-PET prototype using inter-crystal scattering events. Phys Med Biol. (2014) 59:4065–83. 10.1088/0031-9155/59/15/4065 24988897

[26] Lage E Parot V Moore SC Sitek A Udías JM Dave SR, et al. Recovery and normalization of triple coincidences in PET. Med Phys. (2015) 42:1398–410. 10.1118/1.4908226 25735294

[27] Rafecas M Torres I Spanoudaki V McElroy D Ziegler S. Estimating accidental coincidences for pixelated PET detectors and singles list-mode acquisition. Nucl Instrum Methods Phys Res Sect A. (2007) 571:285–8. 10.1016/j.nima.2006.10.084

[28] Torres-Espallardo I Rafecas M Spanoudaki V McElroy DP Ziegler SI. Effect of inter-crystal scatter on estimation methods for random coincidences and subsequent correction. Phys Med Biol. (2008) 53:2391–411. 10.1088/0031-9155/53/9/012 18421120

[29] Oliver JF Rafecas M. MuST, multiples enhanced ST method for randoms rate estimations. In: *2010 IEEE Nuclear Science Symposium Conference Record*. Knoxville, TN: IEEE (2010). p. 3544–7.

[30] Delso G Martinez MJ Torres I Ladebeck R Michel C Nekolla S, et al. Monte Carlo simulations of the count rate performance of a clinical whole-body MR/PET scanner. Med Phys. (2009) 36:4126–35. 10.1118/1.3193676 19810486

[31] Lin H-H Chuang KS Chen SY Jan ML. Recovering the triple coincidence of non-pure positron emitters in preclinical PET. Phys Med Biol. (2016) 61:1904–31. 10.1088/0031-9155/61/5/1904 26878420

[32] Robinson S Julyan PJ Hastings DL Zweit J. Performance of a block detector PET scanner in imaging non-pure positron emitters—modelling and experimental validation with ^124^I. Phys Med Biol. (2004) 49:5505–28. 10.1088/0031-9155/49/24/008 15724539

[33] Moore SC Krishnamoorthy S Blankemeyer E Carlin SD Karp JS Metzler SD. Simultaneous micro-PET imaging of F-18 and I-124 with correction for triple-random coincidences. In: Matej S, Metzler SD, editors. *2019 15th International Meeting on Fully Three-Dimensional Image Reconstruction in Radiology and Nuclear Medicine*. Philadelphia: SPIE (2019). p. 103.

[34] Huang B Li T Ariño-Estrada G Dulski K Shopa RY Moskal P, et al. SPLIT: statistical positronium lifetime image reconstruction via time-thresholding. IEEE Trans Med Imaging. (2024) 43:2148–58. 10.1109/TMI.2024.3357659 38261489 PMC11409919

[35] España S Herraiz JL Vicente E Vaquero JJ Desco M Udias JM. PeneloPET, a Monte Carlo PET simulation tool based on PENELOPE: features and validation. Phys Med Biol. (2009) 54:1723–42. 10.1088/0031-9155/54/6/021 19242053

[36] Abushab KM Herraiz JL Vicente E Cal-Gonzalez J Espana S Vaquero JJ, et al. Evaluation of PeneloPET simulations of biograph PET/CT scanners. IEEE Trans Nucl Sci. (2016) 63:1367–74. 10.1109/TNS.2016.2527789

[37] Lerche CW Niekämper D Scheins JJ Tellmann L Ridder D Kitten Y, et al. First performance results of a UHF-MRI compatible BrainPET insert for neuroscience. In: *2023 IEEE Nucl Sci Symp Conf Rec*. Vancouver: IEEE (2023). p. 1–2.

[38] National Nuclear Data Center. Data from: information extracted from the nudat database (2025). Available online at: https://www.nndc.bnl.gov/nudat/ (Accessed Febuary 26, 2025).

[39] Haemisch Y Frach T Degenhardt C Thon A. Fully digital arrays of silicon photomultipliers (dSiPM)—a scalable alternative to vacuum photomultiplier tubes (PMT). Phys Procedia. (2012) 37:1546–60. 10.1016/j.phpro.2012.03.749

[40] Dadgar M Maebe J Abi Akl M Vervenne B Vandenberghe S. A simulation study of the system characteristics for a long axial FOV PET design based on monolithic BGO flat panels compared with a pixelated LSO cylindrical design. EJNMMI Phys. (2023) 10:75. 10.1186/s40658-023-00593-0 38036794 PMC10689648

[41] Niekämper D Scheins JJ Shah NJ LopezHerraiz J Lerche CW. Precise and accurate coincidence processing for the BrainPET-7T with triple coincidences and randoms. In: *2023 IEEE Nuclear Science Symposium, Medical Imaging Conference and International Symposium on Room-Temperature Semiconductor Detectors (NSS MIC RTSD)*. Vancouver, BC, Canada: IEEE (2023). p. 1.

[42] Pfaehler E Niekämper D Scheins JJ Shah NJ Lerche CW. ML-EM based dual tracer PET image reconstruction with inclusion of prompt gamma attenuation. Phys Med Biol. (2025) 70:015009. 10.1088/1361-6560/ad9660 39577081

[43] Lee MS Kang SK Lee JS. Novel inter-crystal scattering event identification method for PET detectors. Phys Med Biol. (2018) 63:115015. 10.1088/1361-6560/aabe3a 29658493

